# Pathways of allosteric regulation in Hsp70 chaperones

**DOI:** 10.1038/ncomms9308

**Published:** 2015-09-18

**Authors:** Roman Kityk, Markus Vogel, Rainer Schlecht, Bernd Bukau, Matthias P. Mayer

**Affiliations:** 1 Zentrum für Molekulare Biologie der Universität Heidelberg (ZMBH), DKFZ-ZMBH-Alliance, INF282, D-69120 Heidelberg, Germany; 2 Zentrum für Molekulare Biologie der Universität Heidelberg (ZMBH), Deutsches Krebsforschungszentrum, DKFZ-ZMBH-Alliance, INF282, D-69120 Heidelberg, Germany

## Abstract

Central to the protein folding activity of Hsp70 chaperones is their ability to interact with protein substrates in an ATP-controlled manner, which relies on allosteric regulation between their nucleotide-binding (NBD) and substrate-binding domains (SBD). Here we dissect this mechanism by analysing mutant variants of the *Escherichia coli* Hsp70 DnaK blocked at distinct steps of allosteric communication. We show that the SBD inhibits ATPase activity by interacting with the NBD through a highly conserved hydrogen bond network, and define the signal transduction pathway that allows bound substrates to trigger ATP hydrolysis. We identify variants deficient in only one direction of allosteric control and demonstrate that ATP-induced substrate release is more important for chaperone activity than substrate-stimulated ATP hydrolysis. These findings provide evidence of an unexpected dichotomic allostery mechanism in Hsp70 chaperones and provide the basis for a comprehensive mechanical model of allostery in Hsp70s.

## Abstract (web summary)

Hsp70 chaperones are essential for cellular proteostasis, and their function depends on allosteric communication between their nucleotide- and substrate-binding domains. Here, Kityk *et al.* provide a mechanical model of allostery and demonstrate that ATP-induced substrate release is more important for chaperone activity than substrate-stimulated ATP hydrolysis.

## Graphical abstract

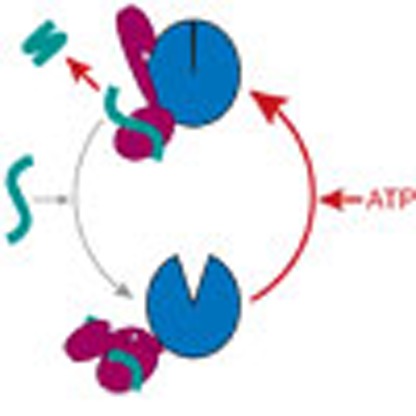

Hsp70 chaperones play an essential role in the entire life of many proteins not only in a stressful environment but also under physiological conditions^1^,^2^,^3^,^4^,^5^,^6^,^7^. The basis for their chaperone activity is the transient interaction of their C-terminal substrate-binding domain (SBD) with short, hydrophobic peptide stretches within their substrate polypeptides^8^. This interaction is regulated through the nucleotide status of the N-terminal nucleotide-binding domain (NBD). In the nucleotide-free and ADP-bound state, the NBD and SBD are largely separated from each other (Fig. 1a; refs 9, 10, 11), only connected by the conserved flexible linker. The two subdomains of the SBD, the two-layered β-sandwich (SBDβ) with the central SB pocket and the α-helical lid domain (SBDα), are packed onto each other, resulting in high affinity for substrates and low substrate association and dissociation rates^12^,^13^,^14^. Upon ATP binding, the two lobes of the NBD rotate relative to each other, SBDα and SBDβ dissociate and dock onto two faces of the NBD (Fig. 1a)^15^,^16^, causing substrate association and dissociation rates to increase by 2 and 3 orders of magnitude, respectively, resulting in low affinity for substrates^12^,^17^,^18^. ATP hydrolysis, which reverts Hsp70 to the high-affinity state, is essential for chaperone function but is extremely slow in the absence of substrates and cochaperones of the J-domain protein family^19^,^20^,^21^,^22^. Binding of a protein substrate to the SBD and a J-domain protein to the NBD synergistically stimulate the ATP hydrolysis rate by >1,000-fold^23^,^24^,^25^,^26^,^27^.

Although aspects of this allosteric control mechanism have been worked out through the effort of many laboratories (for overview see ref. 28), key questions remain unanswered. For example, it is still unclear how substrates stimulate the ATPase activity of Hsp70s. Furthermore, previously identified residues involved in allosteric control always affect both directions of the interdomain communication, the ATP-induced substrate release and the substrate-triggered ATPase activity. Therefore, it is generally believed that the allosteric signals travel in both directions through the same residues of Hsp70 and the importance of each direction of the allosteric control *in vivo* is unclear. To address these questions we analysed the recent crystal structure of *E. coli* DnaK in the ATP-bound open conformation^15^ and studied the defects of single amino acid replacement variants. Our data reveal how the SBD inhibits the ATPase activity and how substrates trigger ATP hydrolysis. We further show that two pathways of the allosteric mechanism can be distinguished, which allowed us to experimentally reveal their importance for chaperone activity *in vitro* and *in vivo*.

## Results

### SBD inhibits ATPase activity through contacts with the NBD

**Figure 1:**
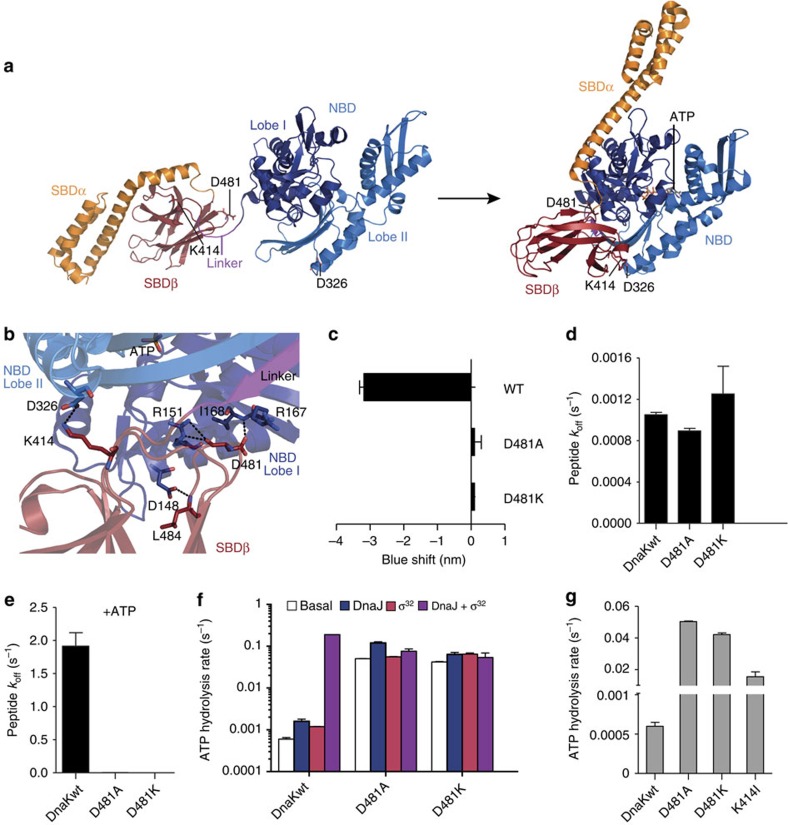
Asp481 is essential for interdomain communication in DnaK. (**a**) Hsp70s alternate between closed and open conformations controlled by nucleotides and substrates through an allosteric mechanism. Cartoon representation of DnaK in the ADP-bound closed conformation (left, PDB ID 2KHO) and in the ATP-bound open conformation (right, 4B9Q^15^). Lobes of the nucleotide-binding domain are coloured in dark blue (I) and light blue (II), SBDβ in dark red and SBDα in orange. The highly conserved interdomain linker is shown in purple. Indicated are residues important for NBD–SBDβ docking. All structure figures were prepared in PyMOL (The PyMOL Molecular Graphics System, Version 1.7.4 Schrödinger, LLC). (**b**) Zoom into the interface between NBD and SBDβ of the ATP-bound open conformation (PDB ID 4B9Q^15^), rotated by 180° as compared with the right panel in **a**. NBD lobes are shown in dark blue and light blue, and SBDβ in dark red and interdomain linker in purple. Residues bridging the domain interface by hydrogen bonds are shown in sticks and coloured according to the atom with carbon in the colour of the respective domain. Putative hydrogen bonds (distances between proton donor and acceptor ≤3.5 Å) are shown as dashed lines. (**c**) ATP-induced blueshift of the emission maximum of typtophane fluorescence. (**d**,**e**) Peptide dissociation rates measured in the absence of nucleotides (**d**) or presence of ATP (**e**). (**f**) Single-turnover ATPase rates in the absence of DnaJ and substrate (basal rate), in the presence of DnaJ, the protein substrate σ^32^ or both DnaJ and σ^32^, as indicated. (**g**) Comparison of basal single-turnover ATPase rates measured in a quenched-flow apparatus for DnaK-D481A, DnaK-D481K and DnaK-K414I. Error bars represent s.e.m. of at least three independent measurements.

The recently solved crystal structure of the ATP-bound open conformation of the *Escherichia coli* Hsp70 protein DnaK^15^,^16^ uncovered an extensive hydrogen bond network in the interface between NBD and SBD (Fig. 1b). A number of residues in this H-bond network, previously identified by genetic screens for functional deficiency or as conserved surface exposed residues, are essential for allostery^10^,^11^,^25^,^29^,^30^,^31^,^32^,^33^,^34^. Since this H-bond network is at the heart of the allosteric control mechanism, each individual residue contributing to it could potentially disclose its so far obscure working principle. One residue, Asp481, which is located in loop L_6,7_ in the SBD, has not been investigated so far, although it is in a central position within this network. Inspection of the atomic structure of DnaK now reveals Asp481 interacts via H-bonds of its side-chain carboxylic group with the backbone amide of Ile168 and of its backbone carbonyl with the guanidine group of Arg151 in lobe I of the NBD. In addition, the interdomain interface may be stabilized by salt bridges between Asp481, Arg167 and Lys155. Arg151, Lys155 and Arg167 were previously shown to be important for interdomain communication^31^.

To probe the contribution of side-chain hydrogen bonds and salt bridge of this potentially critical residue Asp481 to the allosteric control, we replaced it with alanine and lysine (DnaK-D481A and DnaK-D481K). Proficiency in signal transduction between NBD and SBD was assayed by: (1) fluorescence of the single tryptophan 102 situated in the NBD of DnaK, which is indicative for ATP-induced conformational changes in the SBD^35^,^36^,^37^; (2) ATP-induced substrate release; (3) substrate- and DnaJ-mediated stimulation of the single-turnover ATPase rate, avoiding that nucleotide exchange becomes rate-limiting and thereby clouding the effect of DnaJ and substrate.

Upon addition of ATP, the fluorescence emission maximum of wild-type DnaK (DnaKwt) shifts by 3 to 4 nm to the shorter wavelength range (blueshift). In DnaK-D481A and DnaK-D481K the ATP-induced blueshift was completely abolished, suggesting lack of signal transmission from NBD to SBD (Fig. 1c). In the absence of nucleotide, all mutants exhibited substrate dissociation rates similar to DnaKwt (Fig. 1d). Upon addition of ATP, the substrate dissociation rate of DnaKwt was increased 1,800-fold, consistent with published data^12^. In contrast, no substantial stimulation of substrate release was observed for DnaK-D481A and DnaK-D481K (Fig. 1e, Supplementary Fig. 1A). DnaKwt had the expected low basal ATPase rate of 6 × 10^−4^ s^−1^, which was stimulated 2.7- and 2-fold by DnaJ and the DnaK protein substrate σ^32^, the *E. coli* heat shock transcription factor, respectively. The simultaneous presence of DnaJ and σ^32^ led to a >50-fold stimulation of the ATPase rate consistent with the literature^25^,^38^. Both Asp481 variants had a greatly increased basal ATP hydrolysis rates (70- and 84-fold) as compared with DnaKwt (Fig. 1f). These elevated basal ATPase rates were further stimulated by DnaJ 2.4- and 1.5-fold for DnaK-D481A and DnaK-D481K, respectively, but no synergistic stimulation by joint addition of DnaJ and σ^32^ was observed (Fig. 1f, Supplementary Fig. 1B).

The elevated basal ATPase rate of DnaK-D481A and DnaK-D481K suggests that loss of contacts between NBD and SBD suspends the inhibition of ATP hydrolysis in DnaK. Consistently, replacement of the Asp481-interacting residues Arg151, Lys155 and Arg167 by Ala, Lys or Asp also caused an increased basal ATPase rate, *albeit* not to the same extend as in Asp481 replacement variants^30^,^31^. Therefore, we asked whether disruption of NBD–SBDβ contacts at the other end of the NBD–SBDβ interface plane would have similar effects. Lys414 located in loop L_2,3_ in SBDβ forms a hydrogen bond to Asp326 in lobe II of the NBD (Fig. 1b) and was previously described to be important for interdomain communication^29^. We replaced Lys414 with isoleucine and measured the basal single-turnover ATP hydrolysis rate, which had not been determined before, using quenched-flow instrumentation (Fig. 1g). Consistent with our hypothesis, the basal ATPase activity of DnaK-K414I was strongly elevated (26-fold) compared with DnaKwt. This suggests that destabilization of the interdomain interface at the other end of the NBD–SBDβ interface has similar effects on the ATPase activity of DnaK.

Taking together, our data demonstrate that the hydrogen bond network in the interface between NBD and SBD is required for successful signal transmission between the domains and for repression of the ATPase activity of DnaKwt. We also find that individual residues of the H-bond network contribute to very different extents to this repression, revealing the mechanism behind this phenomenon (discussed below).

### Substrate-to-catalytic ATPase centre signal transduction

**Figure 2:**
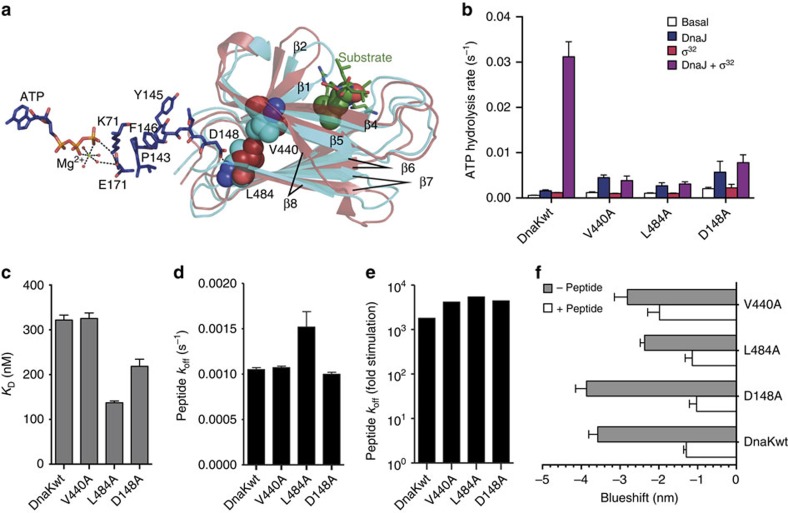
Pathway of substrate stimulation of the ATPase rate. (**a**) Alignment of the SBDβ of the co-crystal structure of DnaK-SBD (cyan, PDB ID 1DKX^60^) with a substrate peptide (green) with the SBDβ (brown) of the crystal structure of DnaK in the ATP-bound open conformation (4B9Q^15^). β1 to β8 indicate the individual β-strands of the two-layered sandwich. Substrate peptide (NRLLLTG) is shown in sticks with the central leucine, which inserts into the hydrophobic substrate-binding pocket of DnaK, in space-filling representation. Residues Val440 and Leu484 in both structures are shown in space-filling representation. Residues that link Leu484 to the ATPase catalytic centre are shown as sticks in atom colours with carbon in dark blue. (**b**) Single-turnover ATPase rates of wild-type and mutant DnaK in the absence of any other protein and in the presence of DnaJ, σ^32^ or both as indicated. (**c**) Dissociation equilibrium constant of wild-type and mutant DnaK for binding of a HiLyte Fluor 488-labelled model peptide (σ^32^-Q132-Q144-C). (**d**) Basal peptide dissociation rates. (**e**) ATP-induced substrate dissociation rates. (**f**) ATP-induced blueshift of the emission maximum of typtophane fluorescence in the absence of a substrate peptide (grey bars) or the presence of 130 μM peptide (σ^32^-Q132-Q144-C). Error bars represent s.e.m. of at least three independent experiments.

Comparison of the structures of the SBD in the ATP-bound open conformation and the isolated SBD with a bound peptide substrate suggests that binding of substrate induces a shift of hydrophobic residues adjacent to the SB pocket, which is propagated through hydrophobic interactions towards the NBD interface and from there to the catalytic centre. In the substrate-bound conformation, Val440, which is on strand 4 adjacent to the substrate interacting Ile438, is shifted away from the SB pocket due to outward movements of strands 3 and 4 of the SBDβ. Val440 has hydrophobic contacts with Leu484, which is shifted closer to the NBD (Fig. 2a, Supplementary Movies 1 and 2 refs 39, 40). Asp148 of the NBD forms a hydrogen bond to the backbone amide nitrogen of Leu484 and could thus transduce any movement in Leu484 through the backbone to Pro143 and further to Lys70 and Glu171, which are involved in catalysis of ATP hydrolysis (Fig. 2a). To verify this signal transduction hypothesis, we replaced Val440, Leu484 and Asp148 by alanine and checked whether the ATPase activities of the mutant proteins are still responsive to stimulation by substrate.

The basal ATPase activities of all three DnaK variants were only slightly elevated (1.5- to 3-fold) as compared with DnaKwt and could be stimulated by DnaJ (Fig. 2b). In contrast, we did not observe any stimulatory effect of σ^32^ on the ATPase activity for any of the variants. Moreover, in the presence of both DnaJ and σ^32^, no synergistic stimulation was observed, and ATP hydrolysis rates were within the experimental error identical to the rates measured in the presence of DnaJ alone. This indicates that the signal transduction from the SBD to NBD is disrupted and the catalytic centre is not ‘sensing' the presence of a substrate. This inability of substrates to stimulate the ATPase activity was not due to a decreased affinity of the DnaK variants for substrates (a fluorescently labelled peptide derived from σ^32^) (Fig. 2c).

The replacement of Val440, Leu484 and Asp148 did not strongly influence signal transduction in the opposite direction—from NBD to SBD: ATP stimulated the wt-like basal substrate dissociation rates to a similar extent as in DnaKwt (Fig. 2d,e). DnaK-D148A exhibited an ATP-induced blueshift of tryptophane fluorescence like DnaKwt. In contrast, for DnaK-V440A and DnaK-L484A the blueshift was reduced by 21% and 34%, respectively. (Fig. 2f). We previously showed that addition of substrate to DnaK·ATP reduced the blueshift of tryptophane fluorescence by 1 to 2 nm before ATP hydrolysis^30^,^31^. We were wondering whether our mutant proteins would show this change in fluorescence. Interestingly, all three mutant proteins exhibited a reduction of the ATP-induced blueshift in the presence of a substrate peptide. Taking together, these results demonstrate that the signal from NBD towards SBD is transduced in DnaK-V440A, DnaK-L484A and DnaK-D148A similar as in DnaKwt, and binding of ATP leads to wt-like conformational changes in the SBD. In contrast, the signal triggering ATP hydrolysis is not transduced from the substrate to the NBD in these DnaK variants.

### Phe146 couples ATP binding to substrate release

**Figure 3:**
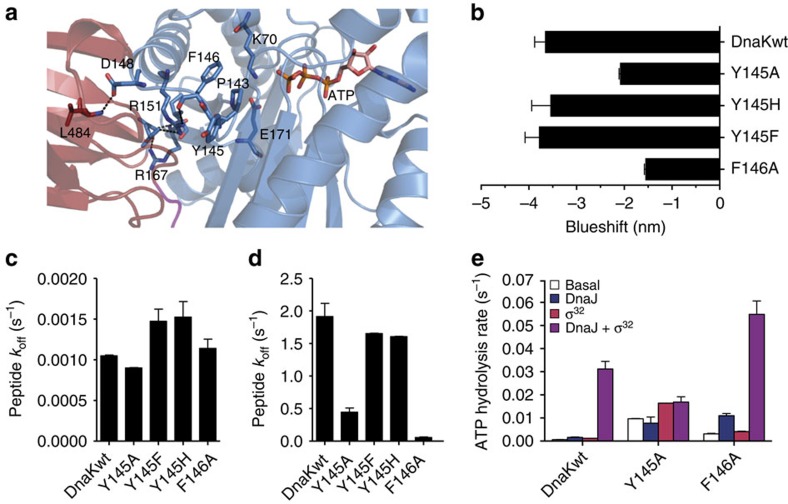
Replacement of Phe146 by alanine compromises signal transduction from ATP to the SBD but not from substrate to the NBD. (**a**) Zoom into the NBD–SBDβ interface of the ATP-bound open conformation of DnaK (PDB ID 4B9Q^15^) with NBD in blue and SBDβ in dark red. ATP and residues involved in catalysis (K70, E171) or allostery (P143, Y145, F146, D148, R151, R167, L484) are shown in sticks in atom colours with carbon in blue (NBD residues) or dark red (SBDβ residues). (**b**) ATP-induced blueshift of the emission maximum of tryptophane fluorescence. (**c**,**d**) Substrate dissociation rates in the absence of nucleotides (**c**) or presence of ATP (**d**). (**e**) Single-turnover ATPase rates of wild-type and mutant DnaK in the absence of any other protein and in the presence of DnaJ, σ^32^ or both as indicated. Error bars represent s.e.m. of at least three independent experiments.

Having discovered that substrate-induced stimulation of the ATPase activity can be uncoupled of ATP-induced release of substrates, we endeavoured to find variants with a phenotype opposite to the variants above, that is, a defect in ATP-induced substrate release but wt-like responsiveness to substrate-induced activation of the ATPase activity. Our attention was drawn to Tyr145 of the NBD, which in both structures of the ATP-bound open conformation (PDB 4B9Q^15^, 4JNE^16^) shows a dramatic change in side-chain conformation as compared with all structures of the isolated NBD (Supplementary Fig. 2A), and to Phe146, which has hydrophobic contacts to Pro143, a residue shown earlier to play an important role in interdomain communication in Hsp70s (Fig. 3a)^30^. Pro143 acts as a switch stabilizing the ATP-bound state, as replacement by alanine or glycine strongly reduces the activation enthalpy for ATP hydrolysis. In fact, Tyr145 and Phe146 together with Val142, Pro143 and Ala144 form a loop that is coordinated and fixed in conformation by two hydrogen bonds to Arg151 (Supplementary Fig. 2A), a residue that also hydrogen bonds to Asp481 in the SBD and was shown to be important for allosteric regulation, as mentioned above^30^. How this ensemble provides the core of the switch between the ATP-bound and ADP-bound conformation is currently unknown. Tyr145 and Phe146 could play an important role in this switch by limiting the flexibility of the loop due to their bulky side chains. Notably, all residues are invariant throughout the Hsp70 family, which suggests an important function.

We analysed the effect of amino acid replacements on allostery as before, by measuring intrinsic fluorescence and ATP-stimulated substrate dissociation. For the conservative Y145F and Y145H replacements, the blueshift was similar to DnaKwt (Fig. 3b). Replacement of Tyr145 and Phe146 by alanine, however, led to a reduction of the blueshift by 43% and 57%, respectively, suggesting some defects in interdomain communication. In the nucleotide-free state, all mutant DnaK variants had very similar substrate dissociation rates as DnaKwt (Fig. 3c). In the presence of ATP, DnaK-Y145F and DnaK-Y145H had also very similar *k*_off_ values as DnaKwt. In contrast, Y145A and F146A replacements resulted in decrease of the peptide dissociation rates to 27% and 3%, respectively, as compared with the rates for DnaKwt (Fig. 3d), indicating a specific defect in allosteric regulation.

To test whether the defect in these two variants affects both directions of interdomain communication, we determined their ATPase activities. DnaK-Y145A and DnaK-F146A had 15- and 5-fold increased basal ATPase rates, respectively (Fig. 3e), which is rather moderate as compared with the above-mentioned DnaK-D481A and DnaK-K414I variants. DnaJ at low concentration (50 nM) had opposing effects on the ATPase activity of the mutant proteins. DnaK-F146A was strongly stimulated by DnaJ alone. In contrast, DnaJ did not stimulate the ATPase rate of DnaK-Y145A. In the absence of DnaJ, σ^32^ stimulated the ATPase activity of DnaK-Y145A and DnaK-F146A by 1.7- and 1.3-fold, respectively. A synergistic stimulation of the ATPase rate by the simultaneous presence of DnaJ and σ^32^ was observed for DnaK-F146A but not for DnaK-Y145A.

These findings indicate that interestingly, the F146A replacement resulted in severe reduction of signal transmission from NBD to SBD, but SBD-to-NBD signal transmission remained largely intact. To exclude that the inability of DnaK-F146A to respond to ATP binding is not simply the consequence of a decreased ATP association rate, we measured the binding kinetics of ATP to DnaK-F146A and DnaKwt (Supplementary Fig. 2B). Under identical conditions, DnaK-F146A bound ATP with apparent single exponential kinetics and an observed association rate of 13.6 s^−1^, while DnaKwt followed biphasic binding kinetics, yielding *k*_fast_=0.31 s^−1^ and *k*_slow_=0.08 s^−1^. Therefore, binding of ATP cannot be rate-limiting for substrate release in DnaK-F146A. In contrast, the lack of a second phase in the association kinetics of this variant is consistent with a defect in the allosteric mechanism since the slower phase observed for DnaKwt has been attributed to the ATP-induced conformational changes.

Taking together, the biochemical analysis indicates that Tyr145 and Phe146 are involved in the allosteric control but with very different functions. Phe146 is important for transmitting the ATP signal to the SBD, while the adjacent Tyr145 couples the signal of the substrate to DnaJ for synergistic stimulation of the ATPase activity.

### Functional implications of defects in allostery

Since we now had at hand DnaK variants that are defective in NBD→SBD signal transmission but proficient in the reverse direction and *vice versa*, we were able to answer the question about the importance of the individual allosteric pathways for chaperone activity and *in vivo* functionality of DnaK.

**Figure 4:**
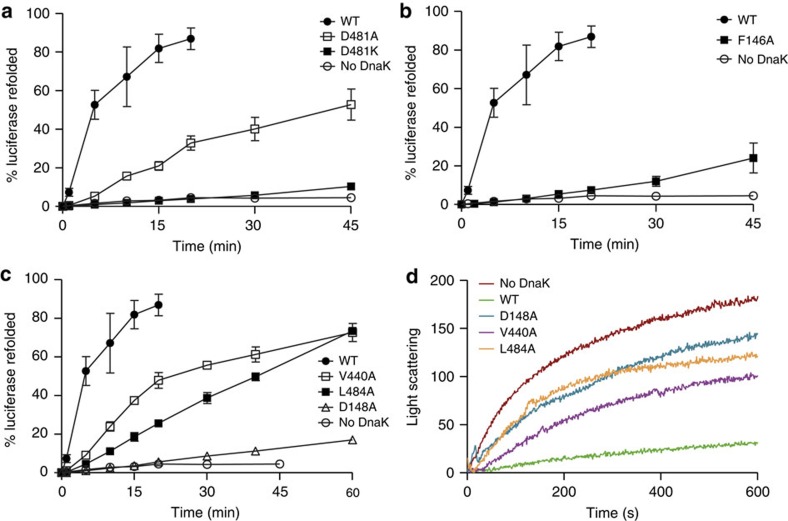
All DnaK variants completely or partially impaired in allosteric regulation had residual chaperone activity *in vitro*. (**a**–**c**) Refolding of chemically denatured luciferase (80 nM) by DnaK (800 nM), DnaJ (160 nM) and GrpE (400 nM). Luciferase was denatured in 6 M guanidinium HCl and diluted 125-fold into refolding buffer containing the respective DnaK variant, DnaJ and GrpE. Shown is the activity of luciferase relative to the not denatured control. Error bars represent s.e.m. of at least three independent experiments. (**d**) Aggregation prevention assay. Guanidinium-denatured luciferase (80 nM) was diluted into a solution containing DnaK (800 nM) and DnaJ (40 nM), and light scattering at 600 nm was followed over time.

To determine the chaperone activity of wild-type and mutant DnaK proteins, we used the established luciferase refolding assay. DnaKwt refolds chemically denatured luciferase to *ca.* 80% within 15 min (Fig. 4a). The completely allosteric-deficient variant DnaK-D481K did not show substantial refolding activity consistent with results for other DnaK variants like DnaK-R151A or DnaK-D393A defective in both signal transmission pathways^30^,^31^. Surprisingly, DnaK-D481A refolded 50% of luciferase in the course of 45 min despite of severe defects in bidirectional allostery.

The NBD*→*SBD variant DnaK-F146A did not refold luciferase efficiently, recovering only 25% of luciferase activity within 45 min (Fig. 4b). The SBD*→*NBD variants DnaK-V440A and DnaK-L484A refolded 75% within 60 min, while DnaK-D148A showed no significant refolding activity (Fig. 4c).

By stimulating the ATPase activity of Hsp70 in synergism with DnaJ, a substrate accelerates the transition of Hsp70 from the low- to the high-affinity state, consequently trapping the substrate itself in the SBD. Thus, we hypothesized that variants that cannot be stimulated by substrates might be less efficient in prevention of protein aggregation. Therefore, we examined whether D148A, V440A and L484A amino acid replacements affect the ability of DnaK to prevent heat-induced aggregation of firefly luciferase in conjunction with DnaJ. In the absence of chaperones, luciferase aggregates within 10 min (Fig. 4d). DnaKwt in the presence of DnaJ was able to suppress the formation of light scattering aggregates. In contrast, DnaK-D148A, DnaK-V440A and DnaK-L484A exhibited reduced prevention of aggregation activity.

*In vivo*, neither *dnaK-D481A* nor *dnaK-D481K* were able to complement the temperature-sensitive growth defect of the Δ*dnaK52::Cm* strain BB1553 (ref. 41) (Table 1). Cells expressing *dnaK-Y145A* from a plasmid did not grow at 40 °C, while *dnaK-Y145F* and *dnaK-Y145H* expression was able to complement the temperature-sensitive growth defect at least partially, underlining the importance of the residue's bulky side chain at that position. Δ*dnaK52::Cm* cells expressing *dnaK-V440A*, *dnaK-L484A* and *dnaK-D148A* from plasmids exhibited much higher plating efficiencies than *dnaK-F146A*. To verify that the difference in plating efficacies is not a consequence of thermal stability of the proteins, we measured circular dichroism spectra of the purified proteins between 10 and 85 °C and determined the temperature-induced unfolding transitions. The measurements yielded two transitions corresponding to the unfolding of NBD and SBD. The melting temperatures of wild-type and mutant proteins were almost identical, indicating that the amino acid replacements did not cause any severe structural defects or impair protein stability (Table 2).

Overall, *in vitro* and *in vivo* functional data are in good agreement with each other. The results suggest that efficient ATP-promoted substrate release from DnaK is more critical for proper functioning than the ability of the substrate to efficiently stimulate the ATPase activity of Hsp70s.

## Discussion

In this study, we gained three important insights into the allosteric mechanism of DnaK, which due to the high conservation within the family is most likely true for all Hsp70 chaperones. (1) Disrupting hydrogen bonds bridging the NBD–SBDβ interface affects the intrinsic ATPase activity of DnaK to very different extents (Asp481, 84-fold; Lys414, 26-fold; Asp148, 3-fold) depending on the position within the interface, indicating that they serve different roles in inhibiting the intrinsic ATPase activity. (2) We reveal the existence of two structurally and functionally distinct signal transmission pathways from NBD to SBD and from SBD to NBD. (3) We show that ATP-induced substrate release is more important for the DnaK chaperone activity *in vivo* than the stimulation of the ATPase activity by substrates.

**Figure 5:**
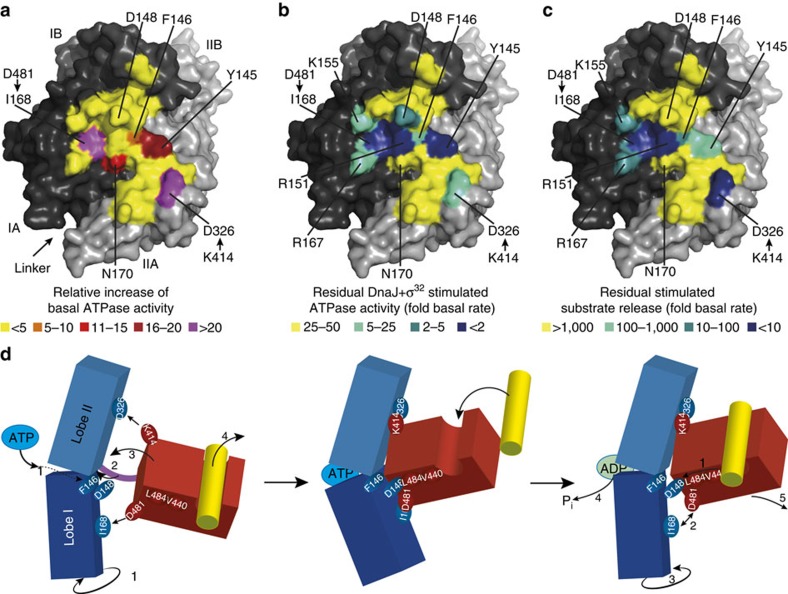
Model of allosteric regulation in Hsp70s. (**a**–**c**) Surface representation of the NBD of DnaK in the ATP-bound open conformation (PDB ID 4B9Q^15^) with lobe I in dark grey, lobe II in light grey and residues interacting with SBDβ coloured according to effects on basal ATPase activity (**a**), on substrate and DnaJ stimulated ATPase activity (**b**), and on ATP stimulated substrate release (**c**), if corresponding residue is replaced by alanine (Y145A, F146A, D148A, R151A, K155A, R167A) or if interacting residue is replaced (I168[D481A], D326[K414I]). (**a**) Fold increase in basal ATPase rate relative to DnaKwt; (**b**) stimulation of ATPase rate by DnaJ plus σ^32^ relative to the basal rate; (**c**) stimulation of substrate release by ATP relative to basal release rates; colouring as indicated. (**d**) Model of allosteric regulation (see main text). For clarity, the SBDα was left away.

What are the mechanistic implications of these insights? Mutations disrupting the interaction between SBDβ and NBD, in particular at the distant points of Asp481/Ile168 and Lys414/Asp326 (Fig. 5a), lead to increased ATPase activity of DnaK, suggesting that the SBDβ keeps the NBD in a conformation incompetent for ATP hydrolysis. When comparing the structure of the isolated NBD (for example, PDB ID 1HPM^42^) with the structure of the NBD of full-length DnaK in the ATP-bound open conformation (PDB ID 4B9Q and 4IPE^15^,^16^) it becomes apparent that ATP binding leads to rotation of lobe I relative to lobe II (Fig. 5d, Supplementary Movie 3 (refs 39, 40)). Such rotation and shearing movements of the NBD subdomains had been deduced earlier from nuclear magnetic resonance (NMR) dipolar coupling experiments of the isolated NBD^43^. It is conceivable that in order to hydrolyze ATP, lobe I has to rotate back to some extent, which is prevented by the interactions of Asp481 with the backbone of Ile168 in lobe I and of Lys414 interacting with Asp326 in lobe II of the NBD. These residues therefore provide the repressing clamp keeping Hsp70 in an ATP state unless activated by DnaJ and substrate. Other residues bridging the NBD–SBDβ interface like Asp148 or Arg151 show a much lower increase of the basal ATPase rate when replaced by alanine (three and fivefold^30^, Fig. 5a), suggesting that they are not in a position to contribute significantly to preventing the back rotation of the NBD lobes. However, they contribute to ATP-induced substrate release and substrate- and DnaJ-mediated stimulation of the ATPase rate (Fig. 5b,c).

Aspartate in position 481 is not universally conserved. In a sequence alignment of 500 Hsp70 sequences of the UniRef90 database only 15% of the sequences have aspartate in this position and 85% asparagine, among which are human Hsp70 and Hsc70 and yeast Ssa proteins. Asparagine also can form a hydrogen bond to the backbone amide of Ile168 similar to aspartate, *albeit* with lower strength. In addition, the electrostatic interactions with Lys155 and Arg167 are lost. Consistently, DnaK-D481N exhibited a slightly reduced frequency of SBDβ to NBD docking in the ATP-bound state as detected by NMR^44^. The precise functional consequences of this are currently unknown but differences in ATPase activity and chaperone function between *E. coli* DnaK and yeast and human Hsp70s have been observed including a lower refolding activity of the denatured model protein luciferase (ref. 45 and unpublished observations).

Although hydrophobic interaction between the NBD and the SBDβ will contribute to stability of the interaction and prevention of back rotation of the NBD lobes, the hydrogen bond between Asp481 and the backbone of Ile168 seems to be critical since it is formed in a hydrophobic environment provided by the side chain of Ile168 and the methylene groups of Arg167 and thus shielded from solvent. This should render this hydrogen bond particularly kinetically stable.

Regarding the signal transduction pathway from the SB pocket to the NBD, our results suggest that SB triggers ATP hydrolysis by pushing through Val440 and Leu484. These residues act as molecular lever on Asp148 in lobe I of the NBD, thereby destabilizing the interaction of Asp481 with the NBD. Thus a very specific series of residues, aligned along a defined pathway starting from the SB pocket, exerts a mechanical force on the trigger Asp148 in the NBD. The prerequisite for such a mechanism to work is the stable anchoring of the SBDβ on the surface of the NBD through hydrogen bonds of Asp481 to Ile168, and Arg151 and Lys414 to Asp326. Disruption of either contact point will disengage the SBDβ and NBD and disrupt substrate stimulation of the ATPase activity, explaining why replacement of Asp481 to alanine or lysine, Lys414 to isoleucine and Arg151 to alanine lead to a loss or significant reduction of substrate stimulation of the ATPase activity^30^.

In a recent NMR study, it was proposed that the conformational dynamics of the SBDβ plays a crucial role in sensing the bound substrate and transmitting the signal to the NBD^46^. As already observed earlier by NMR and by hydrogen exchange experiments, the SBDβ is much more dynamic in the absence of a substrate than when a peptide is bound^47^,^48^,^49^. Based on these observations, it could be hypothesized that the reduced flexibility of the SBDβ upon substrate binding could be the trigger signal that leads to dissociation of the SBDβ from the NBD because it could not accommodate the strain from the interaction, resulting in back rotation of the NBD lobes and ATP hydrolysis. However, such a mechanism would not explain why Asp148-to-Ala exchange abrogates substrate stimulation of the ATPase activity. Such an exchange should not influence the rigidity of the SBDβ in complex with a substrate and should rather weaken the interface between SBDβ and NBD and facilitate SBDβ dissociation, leading to more efficient stimulation of the ATPase activity by substrates. Since this is not observed, it must be concluded that Asp148 is essential for sensing the presence of a substrate in the SB pocket. The side-chain carboxyl group of Asp148 only makes a single polar contact with the backbone amide of Leu484 and is thereby ideally positioned to sense movements of β-strand 7 (Fig. 2a). Since Leu484 and Val440–to-Ala exchanges also abrogate ATPase stimulation by substrates, the side chains of these residues are also necessary for signal transmission. We cannot exclude that there are other residues which would have a similar effect. Extensive study of the different substrate-bound, as well as substrate-free structures did not suggest any other residue that would not at the same time affect substrate binding. DnaK-V440A and DnaK-L484A bind a peptide substrate like DnaKwt (see Fig. 2c) and do not have any defects in any assay we have tested except for the substrate-mediated stimulation of the ATPase activity and a slight reduction of the blueshift of tryptophane fluorescence. The change in conformational dynamics of the SBDβ upon substrate binding might play a role in triggering ATP hydrolysis, however, this cannot be tested directly. In contrast, the residues discovered here are essential for this process.

Intriguingly, substrate was still able to reduce the blueshift of tryptophane fluorescence in these mutant proteins. Structural and biochemical data indicate that the blueshift of tryptophane fluorescence is caused by docking of SBDα to NBD^15^,^16^,^32^,^37^. We previously proposed that the ATP-bound state is dynamic, alternating between an open and closed conformation involving SBDα–NBD docking and undocking^12^,^50^. This hypothesis was attested by single molecule Förster resonance energy transfer and electron paramagnetic resonance experiments, demonstrating different distance distributions between SBDα and SBDβ in the presence of ATP^51^,^52^,^53^. A bound peptide could stabilize the closed conformation and thereby shift the equilibrium between open and closed conformation, explaining the reduced blueshift of tryptophane fluorescence. This is consistent with NMR data, showing that the SBDα undocks from the NBD on peptide binding to DnaK·ATP^44^. DnaK-D148A, DnaK-V440A and DnaK-L484A bind substrates like DnaKwt and might allow latch formation between SBDβ and SBDα, stabilizing the closed conformation even in the absence of a signal to the NBD (Supplementary Fig. 3). These results also indicate that stabilizing the closed conformation is not the signal that triggers ATP hydrolysis.

Our analysis reveals a unique role for residue Phe146 in the allosteric regulation of DnaK. DnaK-F146A has a strongly reduced substrate release rate in the presence of ATP (2.9% DnaKwt), indicating a deficiency in the signal transduction from NBD to SBD. How does Phe146 affect substrate release? Phe146 has no contacts to the SBD and there are no indications that it directly contributes to stabilization of the NBD–SBDβ interface. In addition, once the interface is formed, Phe146 seems to be dispensable because DnaK-F146A is competent for substrate-triggered ATP hydrolysis. The most likely reason for the strongly reduced ATP-stimulated substrate release rate in the DnaK-F146A variant is that Phe146 is important for coupling ATP binding to the rotation of lobe I of the NBD to reach the geometry necessary for docking of the SBDβ. This hypothesis is supported by the fact that the second phase of the ATP association kinetic was not detected. This second phase of ATP association is linked to the ATP-induced conformational change in DnaK^36^. The coupling of ATP binding to lobe I rotation likely occurs through an interaction of the aromatic side chain of Phe146 with Pro143, which was found earlier to act as a switch stabilizing the ATP-bound open conformation^30^, and might contribute to stabilizing its position. Tyr145 seems to contribute to stabilization of Pro143 as well, because the Y145A replacement causes a stronger reduction of the ATP-stimulated substrate release rate than replacements by phenylalanine or histidine. *Albeit*, the replacement of Tyr145 by alanine affects ATP-induced substrate release less than replacement of Phe146 by alanine. However, in contrast to DnaK-F146A, DnaK-Y145A is not responsive to DnaJ, consistent with earlier results^54^ and therefore has other, additional defects. Interestingly, the substrate- and DnaJ-stimulated ATPase rate of DnaK-F146A is 1.8-fold higher than for DnaKwt. On substrate- and DnaJ-induced release of the SBDβ, the NBD lobes rotate back into the ATP hydrolysis competent position. This rotation may occur more freely in DnaK-F146A than in DnaKwt due to the higher flexibility in the Pro143-containing loop, resulting in an elevated ATPase rate.

From these investigations, the following comprehensive mechanical model for the allosteric mechanism of Hsp70 chaperones emerges (Fig. 5d): Incoming ATP works against the bracing (Pro143, F146) to rotate lobe I of the NBD relative to lobe II (left panel, 1). This in turn allows insertion of the linker (connecting the NBD and the SBD) into the lower cleft of the NBD (2) and to bring the mounting (Ile168, Asp326) into the correct position for interaction with the SBDβ (3). The rotating lobes then act on the handles in the SBDβ (Asp481 and Lys414) to snap the SB cleft into the open position and to eject bound substrate (4). These handles in reverse act as a clamp to stabilize the NBD and prevent back rotation and premature ATP hydrolysis. The incoming substrate pushes through the lever (V440, L484) onto the trigger (Asp148) in the NBD (right panel, 1) to release the clamp (2) and allow lobe I back rotation (3) and ATP hydrolysis (4) with concomitant trapping of the substrate, like in a mouse trap, and release of the SBDβ from the NBD (5). This model is congruent with previously published data^15^,^29^,^30^,^31^,^34^,^44^,^55^. In particular, it explains why NBD and SBDβ were found to be largely separated in the simultaneous presence of ATP and substrate in NMR experiments with an ATPase-deficient DnaK variant^44^. This unique allosteric mechanism is generally conserved in evolution, with invariant residues (Pro143, Tyr145, Phe146, Asp148, Arg151) and conservative replacements (168Ile/Val, 326Asp/Asn, 414Lys/Arg, 440Val/Ile, 481Asp/Asn, 484Leu/Val/Ile) at the key positions. The conservative replacements may fine-tune Hsp70 for the requirements of the respective organism or for specific tasks.

Our mutational dissection of the allosteric control mechanism allowed us for the first time to ask the question how important are both arms of the interdomain communication for the chaperone activity of DnaK *in vivo*. The surprising answer is that ATP-stimulated substrate release seems to be more important *in vivo*. The plating efficacy of a strain harbouring DnaK-F146A, which is defective in ATP stimulation of substrate release but proficient in substrate stimulation of the ATPase activity, was significantly lower than for strains harbouring DnaK-V440A, DnaK-L484A or DnaK-D148A with the reverse defect. In addition, in contrast to the situation for DnaK-V440A, DnaK-L484A and DnaK-D148A, higher levels of DnaK-F146A could not compensate for the reduced chaperone activity. This suggests that efficient capture of a substrate without a stiff mechanical coupling of ATP binding to reopening of the SB cleft is more detrimental *in vivo* than inefficient capture of misfolded proteins (see Supplementary Notes). It is possible that the stiff mechanical coupling exerts a force onto the substrate that leads to local unfolding (power stroke) consistent with an earlier proposal that Hsp70s refold proteins by unfolding^56^.

## Methods

### Protein purification

Site-directed mutagenesis was performed by the method by Kunkel *et al.*^57^ as published. Wt (DnaKwt) and mutant DnaK were purified as native proteins, with a C-terminal His-tag or N-terminal His-SUMO fusion after overproduction in *ΔdnaK52* cells (BB1553) to avoid contaminations with DnaKwt. On expression, cell pellets were resuspended in lysis buffer (20 mM Tris/HCl pH 7.9, 100 mM KCl, 1 mM PMSF) and subjected to lysis by Microfluidizer Emulsi Flex C5 (Avestin, Ottawa, Canada) and afterwards applied onto a column with Ni-IDA resin (Macherey-Nagel, Düren, Germany). Subsequently, the column was washed with 20 column volumes of lysis buffer and 10 column volumes of ATP buffer (20 mM Tris/HCl pH 7.9, 100 mM KCl, 5 mM MgCl_2_, 5 mM ATP). The proteins were eluted with elution buffer (20 mM Tris/HCl pH 7.9, 100 mM KCl, 250 mM imidazol). In case of His-SUMO-fused DnaK variants, the proteins were treated with Ulp1 protease to remove the His-SUMO tag from DnaK. On cleavage and dialysis into lysis buffer, the protein mixture was subjected again to a Ni-IDA column and flow-through fraction containing tag-free DnaK was collected. Afterwards, nucleotides bound to DnaK were removed by treating the samples with calf intestine alkaline phosphatase (AP; 2 U AP per mg DnaK) at room temperature overnight on dialysis into the nucleotide removal buffer (50 mM Tris/HCL, pH 7.5, 100 mM KCl, 2 mM EDTA, 2 mM NaN_3_, 2 mM β-mercaptoethanol, 10% glycerol)^36^. To remove AP, DnaK was bound to anion exchange column (Resource Q, GE Healthcare) equilibrated in low salt buffer (40 mM HEPES/KOH pH 7.6, 100 mM KCl, 5 MgCl_2_). DnaK was eluted with a linear KCl gradient (0.1–1 M) within 10 column volumes. The final nucleotide content of the preparations was checked by HPLC^36^ and was generally <1%.

### Kinetic measurements

All kinetic measurements were performed in HKM buffer (25 mM HEPES/KOH, pH 7.6, 50 mM KCl, 5 mM MgCl2) at 30 °C.

*ATPase activity*. The ATPase activities of DnaKwt and DnaK mutant proteins were determined under single-turnover conditions according to ref. 58. The DnaK–ATP complexes (final volume 50 μM) were formed by mixing DnaK (final concentration 30 μM) and 2 μl of ATP-mix (20 mM ATP, 12 μCi [α^32^P]-ATP) in reaction buffer (25 mM HEPES/KOH pH 7.6, 50 mM KCl, 10 mM MgCl_2_) and leaving the mixture for 2 min on ice. The complex was isolated at 4 °C by gel filtration on Sephadex G-50 columns, pre-saturated with 1 ml of a BSA solution (1 mg ml^−1^) and pre-equilibrated with 3 column volumes of the reaction buffer. Twenty fractions (two drops per fraction) were collected. The first four fractions containing radioactivity as detected by Geiger counter were pooled, divided into 6.5 μl aliquots, snap frozen in liquid nitrogen and kept at −80 °C. For the ATPase activity determination, DnaK–ATP complex solutions were thawed in a water bath at 30 °C and, after withdrawal of 0.5 μl for the zero time point, mixed with 44 μl reaction buffer containing either no cofactors or 50 nM DnaJ or 1 μM σ^32^ or both. At given time points, 2 μl were withdrawn from the reaction mixture and spotted onto a thin layer chromatography plate (PEI Cellulose, Merck, Darmstadt, Germany), which was pre-spotted with a mixture of 5 mM ADP and 5 mM ATP. Developing the plates in 400 mM LiCl, 10% acetic acid separated ADP from ATP. Dried plates were exposed to fluoroimaging screens overnight and relative amounts of ATP and ADP were quantified by Fuji FLA 2000 Phosphoimager.

For DnaK-D481A, DnaK-D481K and DnaK-K414I and for DnaKwt in the presence of DnaJ and σ^32^ a quenched-flow apparatus (KinTek RQF-3, KinTek Cooperation, Rochester, NY) was used. DnaK (10 μM) was first mixed with ATP (8 μM) than aged for 1 s and subsequently mixed with HKM buffer without proteins or including DnaJ (1 μM) or σ^32^ (10 μM), or both at 30 °C. Reaction was quenched in 400 mM LiCl, 10% acetic acid and analysed by thin layer chromatography as above.

*Peptide dissociation*. Dissociation rates for peptides were determined, pre-incubating 0.5 μM DnaK and 0.5 μM of the 2-(4′-(iodoacetamido)anilino) naphthalene-6-sulfonic acid (IAANS) labelled peptide σ^32^-Q132-Q144-C (QRKLFFNLRKTKQC)^59^ for at least 30 min before mixing with a 50-fold excess of unlabelled σ^32^-Q132-Q144-C peptide in a Perkin Elmer LS55 Luminescence Spectrometer (*λ*_ex_=335 nm, *λ*_em_=460 nm) in the absence of ATP and a stopped flow instrument (SX.18MV, Applied Photophysics and KinTek SF-300x (KinTek); *λ*_ex_=335 nm, cut-off filter: 420 nm) in the presence of ATP.

*ATP association*. Association of ATP was determined in a stopped flow instrument mixing 2 μM DnaK with 2 μM of the fluorescent analogue N8-(4-N′-methylanthraniloylaminobutyl)-8 aminoadenosine 5′-triphosphate (MABA-ATP, *λ*_ex_=360 nm, cut-off filter: 420 nm)^36^.

### Tryptophane fluorescence

DnaK samples were diluted in HKM buffer to the concentration of 3 μM and tryptophan emission spectra were recorded between 300 and 400 nm in a Perkin Elmer LS55 Luminescence Spectrometer or Jasco FP-6500 Spectrometer (*λ*_ex_=295 nm) in the presence and absence of ATP (2 mM). Peptide σ^32^-Q132-Q144-C at 130 μM was added where indicated.

### Circular dichroism spectroscopy

DnaK variants were dialysed into 10 mM potassium phosphate buffer (pH 7.6) and diluted to the final concentration of 5 μM. The melting curves were obtained by heating up the protein samples from 20 to 85 °C (at rate 0.5°·per min) and recording circular dichroism signal at 222 nm on Jasco J750 spectropolarimeter. To determine the *T*_m_ values, the thermal unfolding equation 
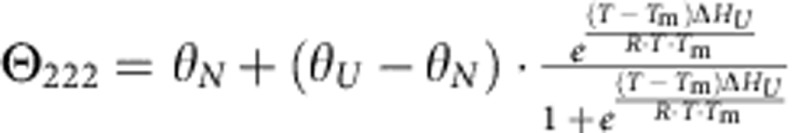
 was fitted to the data. *θ*_N_, ellipticity of the native protein; *θ*_U_, ellipticity of the unfolded protein.

### Luciferase refolding assay

Firefly luciferase (10 μM) was denatured in unfolding buffer (30 mM Tris/acetate pH 7.5, 6 M guanidinium HCl) and diluted 125-fold into refolding buffer (25 mM HEPES/KOH pH 7.6, 100 mM KAc, 10 mM MgAc_2_, 2 mM ATP, 5 mM DTT) (80 nM final luciferase concentration) containing DnaK (800 nM), DnaJ (160 nM) and GrpE (400 nM) at 30 °C. At indicated time points, 1 μl of the reaction mixture was diluted into 124 μl of assay buffer (100 mM K_x_H_y_PO_4_ pH 7.6, 25 mM glycyl-glycine pH 7.4, 100 mM KAc, 15 mM MgAc_2_, 2 mM ATP prepared freshly from stock solutions) and subsequently mixed with 125 μl of luciferin (160 μM) in a Biolumat (Berthold). On mixing, chemical luminescence was measured for 5 s.

### Aggregation prevention of firefly luciferase

Firefly luciferase was denatured in 5 M guanidinium hydrochloride at room temperature for 15 min. Subsequently it was diluted to the final concentration of 80 nM into HKM buffer containing DnaK (800 nM), DnaJ (40 nM), 2 μM ATP, and light scattering at 600 nm at 42 °C was followed over time.

### Substrate affinity determination

The dissociation equilibrium constant for the DnaK–substrate complex was determined by microscale thermophoresis using a fluorescent-labelled peptide. HiLyte Fluor 488-maleimide (Anaspec)-labelled σ^32^-Q132-Q144-C (100 nM) was mixed in HKM buffer containing 0.1% Tween-20 and incubated with increasing concentrations of DnaKwt or its variants (in the range of 0.6 nM–10 μM) at 30 °C for at least 30 min. Afterwards the thermophoresis measurement was performed on Monolith NT.115 instrument (Nanotemper) (blue filter, LED power 40%, MST power 80%, 30 °C).

### Complementation assay

Δ*dnaK52 E. coli* strain (BB1553; ref. 41), containing *lacI*^*q*^-expressing plasmid pDMI,1, was transformed with plasmids containing IPTG-inducible *dnaK* alleles harbouring mutations-of-interest. Ten fold dilutions of an overnight culture were spotted onto Luria-broth plates containing isopropyl β-D-1-thiogalactopyranoside (0–250 μM). The plates were subsequently incubated overnight at 30 and 40 °C.

## Additional information

**How to cite this article:** Kityk, R. *et al.* Pathways of allosteric regulation in Hsp70 chaperones. *Nat. Commun.* 6:8308 doi: 10.1038/ncomms9308 (2015).

## Supplementary Material

Supplementary Information Supplementary Figures 1-3, Supplementary Note and Supplementary References

Supplementary Movie 1 Supplementary Movie 1 (related to Fig. 2A): Movements of Val440 and Leu484 during transition from the open to the substrate bound conformation. The movie shows a morph of the SBDβ in cartoon representation starting from the structure of the ATP bound open conformation of E. coli DnaK (PDB ID 4B9Q) morphing into the structure of the isolated SBD of E. coli DnaK in complex with a peptide (PDB ID 1DKX and back (side view; L_7,8_ and strain 8 left away for clarity). In space filling representation are shown residues Leu484 (left bottom) and Val440 (middle top), and the residues Phe426 and Ile438, which line the substrate binding pocket (right). For residues Val440, Phe426, and Ile438 only Cα, and side chain atoms are shown. Morph was created on The Yale Morph Server (http://molmovdb.org/morph/) and visualized in PyMOL (The PyMOL Molecular Graphics System, Version 1.7.4 Schrödinger, LLC).

Supplementary Movie 2 Supplementary Movie 2 (related to Fig. 2A): Movements of Val440 and Leu484 during transition from the open to the substrate bound conformation. The movie shows a morph of the SBDβ in cartoon representation starting from the structure of the ATP bound open conformation of E. coli DnaK (PDB ID 4B9Q) morphing into the structure of the isolated SBD of E. coli DnaK in complex with a peptide (PDB ID 1DKX and back (top view; L_7,8_ and strain 8 left away for clarity). In space filling representation are shown residues Leu484 (left) and Val440 (middle), and the residues Phe426 and Ile438, which line the substrate binding pocket (right). For residues Val440, Phe426, and Ile438 only Cα, and side chain atoms are shown. Morph was created on The Yale Morph Server (http://molmovdb.org/morph/) and visualized in PyMOL (The PyMOL Molecular Graphics System, Version 1.7.4 Schrödinger, LLC).

Supplementary Movie 3 Supplementary Movie 3 (related to Fig. 5): ATP induced dynamics of the NBD. The movie shows a morph starting from an homology model of E. coli DnaK onto the structure of bovine Hsc70 in complex with ADP and phosphate (PDB ID 1HPM) to the ATP bound open conformation of DnaK (PDB ID 4B9Q) and back. Surface representation with lobe I in dark gray, lobe II in light gray, and residues interacting with SBDβ colored according effects on basal ATPase rate upon replacement with alanine (Y145A, F146A, D148A, R151A, K155A, R167A) or upon replacement of interacting residues (I168[D481A], D326[K414I]). Increase in basal ATPase rate less than 5-fold (yellow). 5 to 10-fold (orange), 11 to 15-fold (red), 16 to 20-fold (dark red), more than 26 to 84-fold (magenta). Morph was created on The Yale Morph Server (http://molmovdb.org/morph/) and visualized in PyMOL (The PyMOL Molecular Graphics System, Version 1.7.4 Schrödinger, LLC).

## Figures and Tables

**Table 1:** *In vivo* phenotype of the mutant *dnaK* alleles.

	**IPTG (μM)**				
	**0**	**50**	**100**	**150**	**250**
*dnaKwt*	—	0.06	1.18	0.67	0.76
Vector	—	—	—	—	—
*dnaK-*Y145F	—	—	0.0005	0.26	0.68
*dnaK-*Y145H	—	—	0.97	0.8	0.002
*dnaK-*Y145A	—	—	—	—	—
*dnaK-*F146A	—	0.0001	0.045	0.09	0.0002
*dnaK*-D481A	—	—	—	—	—
*dnaK*-D481K	—	—	—	—	—
*dnaK*-V440A	—	0.048	0.69	0.80	0.90
*dnaK*-L484A	—	0.0005	0.048	0.90	0.95
*dnaK*-D148A	—	0.0001	0.15	0.67	0.75

IPTG, isopropyl β-D-1-thiogalactopyranoside.

Plating efficiency at 40 °C divided by plating efficiency at 30 °C; —, plating efficiency <10^−4^.

**Table 2:** Stability of the mutant proteins is not severely compromised by the mutational alterations.

	***T***_**m,1**_ **(°C)**	***T***_**m,2**_ **(°C)**
DnaKwt	41.6±0.2	75.5±0.3
DnaK-Y145F	46.5±0.9	77.7±1.0
DnaK-Y145H	47.2±0.3	74.4±0.4
DnaK-Y145A	47.6±0.1	74.5±0.1
DnaK-F146A	42.0±0.1	74.9±0.2
DnaK-D148A	40.6±0.5	72.1±0.3
DnaK-V440A	45.6±0.3	72.7±0.8
DnaK-D481A	43.3±0.2	72.0±0.2
DnaK-D481K	43.6±0.2	72.6±0.4
DnaK-L484A	47.0±2.0	72.7±0.4

*T*_m_ was determined by fitting the thermal unfolding equation to circular dichroism spectroscopy traces at 222 nm.
