# Supplementary material for: Pathways of allosteric regulation in Hsp70 chaperones
Source: Nat Commun. 2015 Sep 18;6:8308. doi: 10.1038/ncomms9308 (PMC4595643; doi:10.1038/ncomms9308)
Supplement: Supplementary Information — Supplementary Figures 1-3, Supplementary Note and Supplementary References [file ncomms9308-s1.pdf]

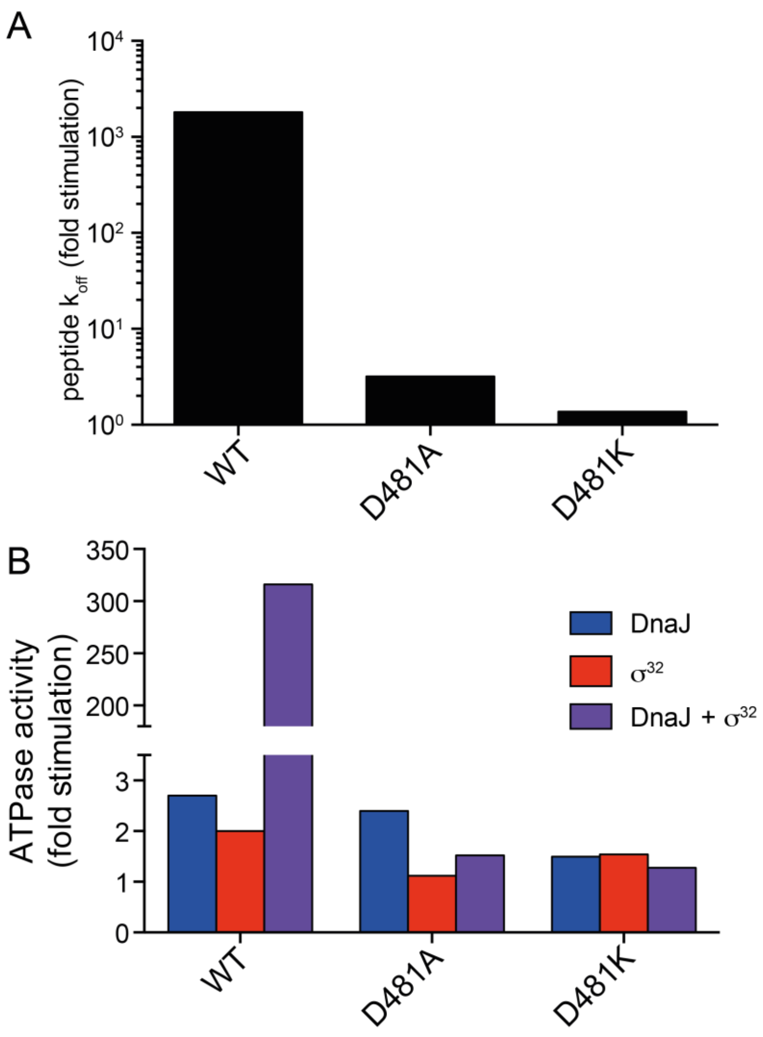

Supplementary Fig. 1 (related to Fig. 1): Asp481 in DnaK is essential for interdomain communication. **A**, Substrate release in amino acid replacement variants of Asp481 in DnaK is stimulated by 1.4 and 3.2-fold as compared to >1000-fold stimulation of wild-type DnaK. **B**, The ATPase activity of DnaK-D481A and D481K is not stimulated synergistically by DnaJ and σ^{32}. Fold stimulation of the ATPase activity relative to the intrinsic ATPase rate.

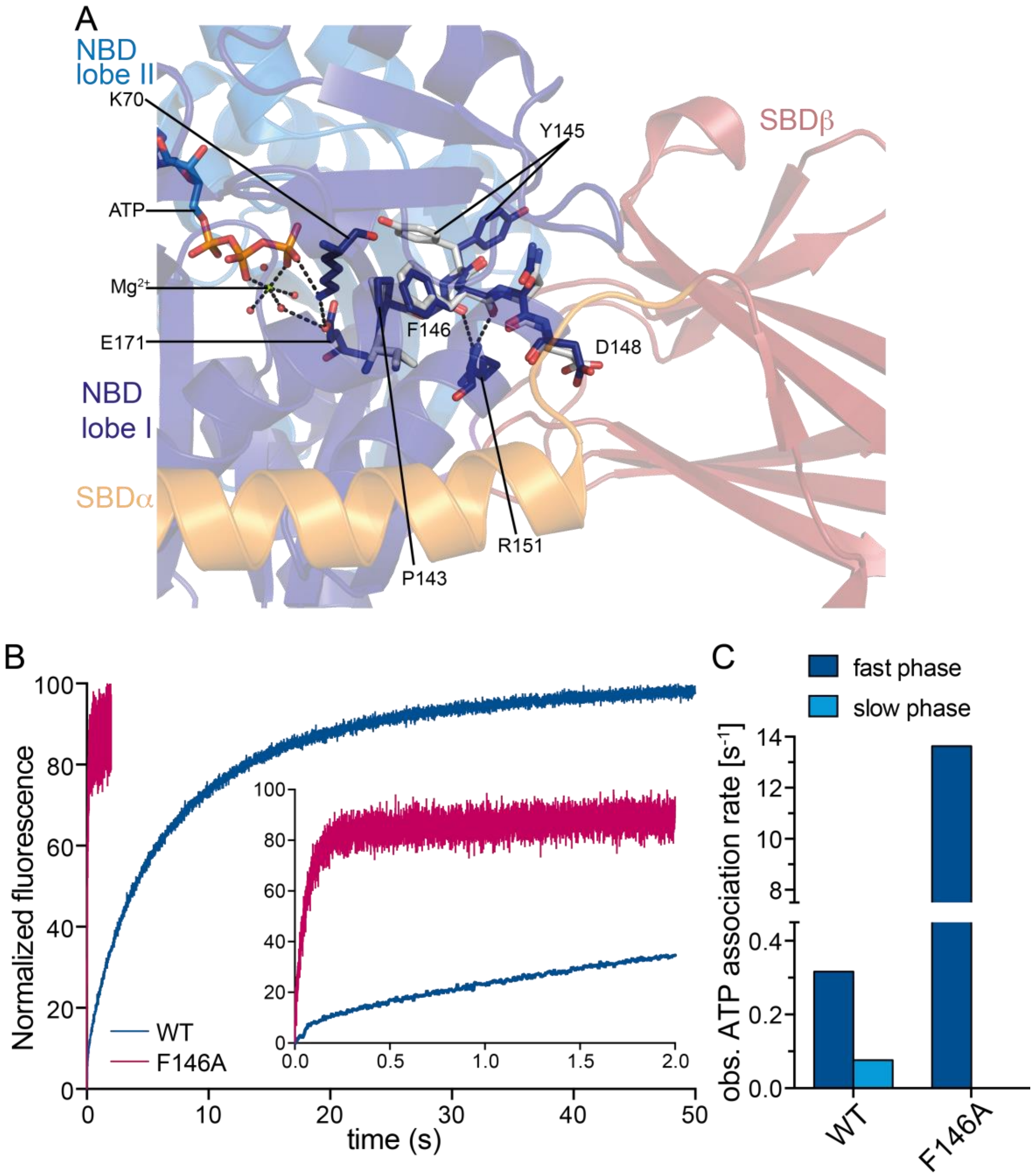

Supplementary Fig. 2 (related to Fig. 3): DnaK-F146A is not deficient in ATP binding. **A**, Zoom into the NBD-SBDβ-interface of the crystal structure of the ATP bound open conformation of DnaK (PDB ID 4B9Q, [1]). NBD colored in blue, SBDβ, brown, SBDα, orange; ATP and selected residues involved in catalysis (K70, E171) or allosteric regulation investigated earlier (P143, R151, [2]) or in this study (Y145, F146, D148) are shown as sticks and colored according to the atom characteristic with carbon in light blue. Residues V142 to D148 are aligned with a homology model of DnaK onto the structure or bovin Hsc70 NBD crystallized in the presence of ADP and phosphate (PDB ID 1HPM, [3]) colored in atom colors with carbon in white as a representative to all structures of the NBD in different nucleotide states. Note the orientation of the side chain of Tyr145. **B**, Association kinetics of MABA-ATP to DnaKwt (blue) and DnaK-F146A (red); inset shows a zoom into the first 2 s. **C**, observed ATP association rates determined by fitting traces in B with single and bi-exponential functions.

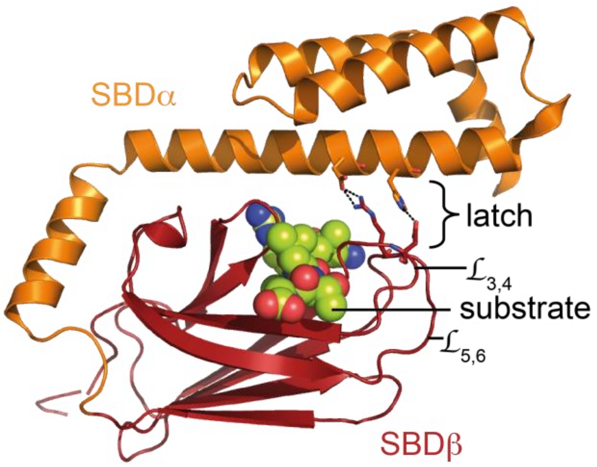

Supplementary Fig. 3 (related to Fig. 2F): **The latch stabilizes the closed conformation of the SBD**. Cartoon representation of the SBD of DnaK (PDB ID 1DKX, [4]. SBDβ colored in dark red; SBDα in orange; substrate in space-filling representation in atom colors with carbons in green. Residues forming hydrogen bonds between SBDα helix B and outer loops $L_{3,4}$ and $L_{5,6}$, the so-called latch, as sticks in atom colors with carbons in the color of the respective subdomain.

Supplementary Notes

Consequences of interrupting bi-directional interdomain communication by Asp481 replacement for chaperone activity

The analysis of the *in vitro* and *in vivo* chaperone activity of the mutant proteins yielded surprising results. DnaK variants defective in allosteric regulation described so far were also found devoid in chaperone activity [2,5-9]. In contrast, we find here that DnaK-D481A, which is completely deficient in interdomain communication, nevertheless refolded chemically denatured luciferase, albeit at a lower rate than DnaKwt. The very high basal ATPase rate of this variant might allow it to capture misfolded luciferase efficiently even in the absence of substrate stimulation of the ATPase activity. In contrast, DnaK-D481K did not yield significant amounts of refolded luciferase. Obviously, the positive charge in this position has a detrimental effect. Asp481 is in close proximity to Lys155 and Arg167 in the ATP bound structure of DnaK, suggesting electrostatic interactions. A positive charge would thus further weaken the NBD-SBDβ interface. In addition, Arg167 was found to be important for interaction with DnaJ [10]. Therefore, a positive charge in position 481 would also affect interaction with DnaJ. This hypothesis is supported by the fact that DnaJ stimulated the ATPase activity of DnaK-D481A more effectively than the ATPase activity of

DnaK-D481K. Therefore, DnaJ-mediated delivery of luciferase to DnaK might still be operative for DnaK-D481A but not for DnaK-D481K. In these *in vitro* refolding assays of chemically denatured luciferase however, DnaJ is present at a two-fold molar excess over luciferase, which facilitates its role in preventing luciferase aggregation [11] and its transfer onto DnaK. In contrast, *in vivo* neither of the two DnaK-D481 variants was able to complement the temperature sensitive phenotype of Δ*dnaK52* mutants (Table 1). The reason for this is most likely the very low DnaJ concentration in the Δ*dnaK52* strain, in which the promoter of the *dnaK-dnaJ* operon is deleted together with the 5' half of the *dnaK* coding sequence [12].

Consequences of interrupting interdomain communication from SBD to NBD for chaperone activity

The DnaK variants that were deficient in SBD to NBD allosteric regulation but proficient in the reverse direction (DnaK-V440A, DnaK-L484A) also exhibited significant refolding activity *in vitro*. This observation cannot be explained by a strongly elevated basal ATPase rate as in the case of DnaK-D481A because their basal ATPase rate was only increased 2 to 3-fold instead of 85-fold. Since substrates do not stimulate the ATPase activity in these variants, their major defect is the lower efficiency of capturing misfolded proteins and preventing aggregation. This was demonstrated in an aggregation prevention assay (Fig. 4D). The important difference between aggregation prevention assays and refolding assays is the DnaJ concentration. Aggregation prevention assays are carried out at DnaJ concentrations that are only half of the substrate concentration. At this concentration DnaJ is not able to prevent aggregation by itself and depends on efficient capture of substrates by DnaK. In contrast, in luciferase refolding assays DnaJ is at concentrations twofold higher than luciferase, which allows DnaJ to completely prevent luciferase aggregation. This explains the refolding activity of these DnaK variants because capturing the misfolded protein is not a limiting step under such conditions. *In vivo*, prevention of aggregation might not be so important due to the highly efficient disaggregation machinery; ClpB and DnaK-mediated solubilization of protein aggregates and subsequent transfer to the DnaK system for refolding might not depend to a similar degree on substrate-mediated stimulation of the ATPase activity. Therefore, DnaK-variants that are not able to prevent aggregation can nevertheless complement the temperature sensitivity phenotype of the ΔdnaK52 strain.

Supplementary References

1. Kityk, R., Kopp, J., Sinning, I. & Mayer, M. P. Structure and dynamics of the ATP-bound open conformation of Hsp70 chaperones. *Molecular Cell* **48,** 863–874 (2012).
2. Vogel, M., Bukau, B. & Mayer, M. P. Allosteric regulation of Hsp70 chaperones by a proline switch. *Molecular Cell* **21,** 359–367 (2006).
3. Wilbanks, S. M. & McKay, D. B. How potassium affects the activity of the molecular chaperone Hsc70. II. Potassium binds specifically in the ATPase active site. *J Biol Chem* **270,** 2251–2257 (1995).
4. Zhu, X. *et al.* Structural analysis of substrate binding by the molecular chaperone DnaK. *Science* **272,** 1606–1614 (1996).
5. Montgomery, D. L., Morimoto, R. I. & Gierasch, L. M. Mutations in the substrate binding domain of the Escherichia coli 70 kDa molecular chaperone, DnaK, which alter substrate affinity or interdomain coupling. *Journal of Molecular Biology* **286,** 915–932 (1999).
6. Laufen, T. *et al.* Mechanism of regulation of hsp70 chaperones by DnaJ cochaperones. *Proc Natl Acad Sci USA* **96,** 5452–5457 (1999).
7. Vogel, M., Mayer, M. P. & Bukau, B. Allosteric regulation of Hsp70 chaperones involves a conserved interdomain linker. *J Biol Chem* **281,** 38705–38711 (2006).
8. Smock, R. G. *et al.* An interdomain sector mediating allostery in Hsp70 molecular chaperones. *Mol. Syst. Biol.* **6,** 414 (2010).
9. Kumar, D. P. *et al.* The four hydrophobic residues on the Hsp70 inter-domain linker have two distinct roles. *Journal of Molecular Biology* **411,** 1099–1113 (2011).
10. Suh, W. C. *et al.* Interaction of the Hsp70 molecular chaperone, DnaK, with its cochaperone DnaJ. *Proc Natl Acad Sci USA* **95,** 15223–15228 (1998).
11. Schröder, H., Langer, T., Hartl, F. U. & Bukau, B. DnaK, DnaJ and GrpE form a cellular chaperone machinery capable of repairing heat-induced protein damage. *EMBO J* **12,** 4137–4144 (1993).
12. Paek, K. H. & Walker, G. C. Escherichia coli dnaK null mutants are inviable at high temperature. *Journal of Bacteriology* **169,** 283–290 (1987).
